# Head and neck tumor segmentation convolutional neural network robust to missing PET/CT modalities using channel dropout

**DOI:** 10.1088/1361-6560/accac9

**Published:** 2023-04-25

**Authors:** Lin-mei Zhao, Helen Zhang, Daniel D Kim, Kanchan Ghimire, Rong Hu, Daniel C Kargilis, Lei Tang, Shujuan Meng, Quan Chen, Wei-hua Liao, Harrison Bai, Zhicheng Jiao, Xue Feng

**Affiliations:** 1 National Engineering Research Center of Personalized Diagnostic and Therapeutic Technology, Hunan, 410008, People’s Republic of China; 2 Department of Radiology, Xiangya Hospital, Central South University, Hunan, 410008, People’s Republic of China; 3 Department of Radiology and Radiological Science, Johns Hopkins Medicine, Baltimore, MD, 21287, United States of America; 4 Department of Radiology, Brown University, Providence, RI, 02903, United States of America; 5 Carina Medical, Lexington, KY, 40513, United States of America; 6 Department of Neurology, Xiangya Hospital, Hunan, 410008, People’s Republic of China; 7 Department of Radiation Medicine, University of Kentucky, Lexington, KY, 40535, United States of America

**Keywords:** head and neck tumor segmentation, 3D UNet, channel dropout, ensemble, deep learning

## Abstract

*Objective*. Radiation therapy for head and neck (H&N) cancer relies on accurate segmentation of the primary tumor. A robust, accurate, and automated gross tumor volume segmentation method is warranted for H&N cancer therapeutic management. The purpose of this study is to develop a novel deep learning segmentation model for H&N cancer based on independent and combined CT and FDG-PET modalities. *Approach*. In this study, we developed a robust deep learning-based model leveraging information from both CT and PET. We implemented a 3D U-Net architecture with 5 levels of encoding and decoding, computing model loss through deep supervision. We used a channel dropout technique to emulate different combinations of input modalities. This technique prevents potential performance issues when only one modality is available, increasing model robustness. We implemented ensemble modeling by combining two types of convolutions with differing receptive fields, conventional and dilated, to improve capture of both fine details and global information. *Main Results*. Our proposed methods yielded promising results, with a Dice similarity coefficient (DSC) of 0.802 when deployed on combined CT and PET, DSC of 0.610 when deployed on CT, and DSC of 0.750 when deployed on PET. *Significance*. Application of a channel dropout method allowed for a single model to achieve high performance when deployed on either single modality images (CT or PET) or combined modality images (CT and PET). The presented segmentation techniques are clinically relevant to applications where images from a certain modality might not always be available.

## Introduction

Radiation therapy is a primary treatment method for head and neck (H&N) cancer. In radiation therapy planning, accurate delineation of target volume is one of the most important and challenging steps (Zhong *et al* 2021). However, reliance on manual annotation of target volume and lesion delineation is accompanied by great challenges such as significant human labor requirements, potential bias, and inter-observer variability (IOV) (Andrearczyk *et al* 2020). A study assessed IOV in delineation of HNSCC (head and neck squamous cell carcinoma) gross tumor volume (GTV), obtaining inter-observer consensus Dice similarity coefficients (DSC) of 0.57 and 0.69 on CT and PET-CT respectively (Gudi *et al* 2017). This highlights the difficulty of consistent manual segmentation, even for experienced oncologists. Automatic segmentation allows for faster, less error-prone, and reproducible delineation of lesions with important clinical relevance (Sharma and Aggarwal 2010, Dou *et al* 2017). In recent times, the development of deep learning-based methods using convolutional neural networks (CNN) has made effective and accurate semantic segmentation possible (Lecun *et al* 2015, Girshick 2015, Long *et al* 2015). Recent studies applying such methods to lesion segmentation have demonstrated the medical applicability of CNNs (Brosch *et al* 2016, Jafari *et al* 2016, Bellver *et al* 2017).

Metabolic and anatomical information from F-FluoroDeoxyGlucose (FDG)-positron emission tomography (PET) and computed tomography (CT) are significant in disease characterization and staging in H&N cancer (Langer 2010, Lewis-Jones *et al* 2016, Goel *et al* 2017, Send *et al* 2017, Zhao *et al* 2018, Moe *et al* 2019). Combination of these modalities has additional potential to improve segmentation and clinical value (Blanc-Durand *et al* 2018, Zhao *et al* 2018, Kawauchi *et al* 2020, Kumar *et al* 2020, Li *et al* 2020, Protonotarios *et al* 2022). Nevertheless, few approaches have reported their feasibility in automatic segmentation tasks for head and neck cancer (Song *et al* 2013, Blanc-Durand *et al* 2018, Moe *et al* 2019).

A major issue in clinical practice when leveraging multi-modalities is modality drop or unusability due to severe artifacts, such as those caused by metallic implants, motion, and contrast medium (Sureshbabu and Mawlawi 2005, Blodgett *et al* 2011, Van Den Wyngaert *et al* 2020). Since CT is used for attenuation correction of PET data, artifacts typically manifest in PET scans; for example, a study found that misregistration in cardiac PET/CT scans caused PET artifact in 40% of patients (Gould *et al* 2007). Another study detected PET/CT misalignment in >60% of cardiac PET-CT studies (Lautamäki *et al* 2008). One immediate solution is to train a network for each modality separately (3D CT, 3D PET), as well as a network for combined modalities. However, this approach increases deployment complexity, especially when there are additional modalities since one CNN is needed for every possible combination. In this case, we propose the simple and robust implementation of a channel dropout technique to emulate different combinations of input modalities, preventing the network from learning the co-adaptation of different input sources without requiring structural change. Therefore, a single network can be trained to accommodate variability of available input modalities in deployment.

Our technique borrows concepts from a commonly-used technique to prevent overfitting in neural networks (Hinton *et al* 2012, Srivastava *et al* 2014). Traditionally, dropout sets random weights within the network to zero to prevent complex co-adaptations between model weights (Hussain *et al* 2018). Similarly, in our proposed channel dropout technique, we randomly drop one of the input imaging modalities (either 3D CT or 3D PET) during training. This can prevent the network from developing co-adaptation of different modalities and allow it to accurately segment the tumor region despite potentially missing one or more modalities.

There have been various previous studies that address missing modalities. Li *et al* used random modality voxel dropout (RMVD) for intervertebral disc localization and segmentation where they randomly zeroed out a subset of voxels in a randomly selected imaging modality during training (Li *et al* 2018). Feng *et al* implemented a similar approach for multiple sclerosis lesion segmentation on multi-sequence magnetic resonance imaging (MRI) but instead randomly zeroed out voxels from entire sequences during training (Feng *et al* 2018). Other studies have employed similar approaches to address missing modalities, but differ in methodology (e.g. scaling remaining inputs after dropout, multiple channel drop, guaranteed dropout of a number of input channels) and accompanying network (van Garderen *et al* 2019, Lau *et al* 2019, La Rosa *et al* 2020). To our knowledge, the generalizability of dropout has mostly been studied on MRI and its applicability to PET and CT imaging are not well described in the literature. Assumption that channel dropout on PET and CT imaging will be successful cannot be guaranteed as models may require the synergistic information provided by both modalities to accurately segment region of interest (ROI).

## Methods

Our data pipeline for automated segmentation of H&N tumors involves the following steps: preprocessing of CT and PET imaging, patch extraction, training of individual models, generating model predictions, ensemble modeling, and image postprocessing. Further information on each methodology is outlined below.

### CT/PET image pre-processing

The primary focus of image pre-processing was to fuse information from CT and PET. For CT images, intensity values were clipped to −1000 and 600 Hounsfield units (HU) to focus the intensity window to soft tissue, given that bone is generally greater than 700 on the Hounsfield scale (Bibb *et al* 2015). Then, each image (PET or CT) was normalized by subtracting the intensity values by their mean and dividing by standard deviation.

To reduce the imaging field-of-view (FOV), each image was cropped based on anatomy to include only the head and neck region. Isotropic resampling by linear interpolation to 1 × 1 × 1 mm^3^ was implemented to ensure consistent voxel spacing across CT and PET images (Parker *et al* 1983). Considering that average voxel sizes of the original CT and PET images were 1.1 × 1.1 × 2.7 mm^3^ and 3.9 × 3.9 × 3.7 mm^3^, respectively, PET images were upsampled to a greater degree after resampling. The sizes of the PET and CT images for a patient were similar but not identical after resampling. Therefore, the larger image (either PET or CT) was cropped to the overlapping field-of-view of the smaller image, using the origin from image metadata as the reference point. The cropped, resampled PET and CT images were then concatenated to generate a 4D input image.

### Patch extraction

Patch-based segmentation of size 128 × 128 × 128 was used to overcome challenges (large memory requirement, long training time, tumor versus non-tumor class imbalance) associated with training 3D U-Net models on large images. For context of the size of the patch with respect to the whole image, the mean size of the CT and PET images after resampling to isotropic resolution was 540 × 540 × 380 and 531 × 531 × 578 voxels, respectively. Considering that the ROI is much smaller than the whole image, the model may not see enough tumor regions during training to accurately identify the ROI after deployment. Therefore, during patch selection, oversampling of the ROI was performed by ensuring at least 50% of training patches included tumor voxels. Patch selection functioned as a built-in method of data augmentation, as variations in randomly selected patches occurred throughout training. Additional data augmentation techniques included horizontal flip (left and right), scaling, brightness, resolution, contrast, and rotation transformations (Hussain *et al* 2017). Specifically, patches were horizontally flipped 50% of the time to take advantage of the head and neck region being anatomically symmetric along the left–right direction. Patches were randomly scaled 20% of the time by a random factor between 0.7 and 1.4, adjusted for brightness 15% of the time by increasing the normalized intensities by a random factor between 0.75 and 1.25, and adjusted for contrast 15% of the time by multiplying the normalized intensities by a random factor between 0.75 and 1.25. Resolution transformations were performed by adding Gaussian noise to the patch 10% of the time. Finally, patches were randomly rotated up to ±30 degrees 20% of the time. To avoid producing border artifacts from data augmentation, patches were selected to be slightly larger than required for network input. After performing data augmentation, a center crop was applied on the augmented images to match the patch size required by the network for training.

### U-Net Structure and training

We implemented a 3D U-Net architecture with 5 levels of encoding and decoding. Encoding blocks included the reduction of image dimensions by a factor of 2, and decoding blocks included 3D transposed convolutions with skip-connections to parallel encoding blocks (Ronneberger *et al* 2015). Consecutive to each encoding-decoding block were layers, for instance, normalization and activation by leaky rectified linear unit (ReLU). Each decoding block had a separate 1 × 1 convolution to generate an intermediate segmentation output. Model loss was computed by summing Dice loss and cross-entropy through the technique of ‘deep supervision’, in which loss was calculated after each decoding block (starting with the second block) and weighted losses were added together to produce a total loss, allowing for training along each layer of the network (Isensee *et al* 2021). Formally, for each decoding block (starting with the second block), let ${o}_{i}\in [0,1]$ be the intermediate segmentation output of the decoding block $i,$ and ${y}_{i}\in [0,1]$ be the corresponding label. Note that at each decoding block output, the corresponding label was produced by down-sampling the ground-truth segmentation mask/labels. Cross entropy (CE) and Dice (D) losses were calculated as below

\begin{eqnarray*}\mathrm{CE}=\,\displaystyle \sum _{i}{y}_{i}\,\mathrm{log}\left({o}_{i}\right)+\left(1-y\right)\mathrm{log}(1-{o}_{i})\end{eqnarray*}

\begin{eqnarray*}{\mathrm{D}}=\,-\frac{2{\sum }_{i}{o}_{i}{y}_{i}}{{\sum }_{i}{o}_{i}+{\sum }_{i}{o}_{i}}.\end{eqnarray*}

Thus, for each decoding block (starting with the second block), loss = $\mathrm{CE}+{\mathrm{D}}.$ The final loss was the sum of the weighted averages of loss from each participating decoding block:

\begin{eqnarray*}\mathrm{Final}\,\mathrm{loss}\,=\,{w}_{1}{L}_{1}+{w}_{2}{L}_{2}+{w}_{3}{L}_{3}+{w}_{4}{L}_{4}\end{eqnarray*}

Here, ${L}_{1}$ would be the last decoding block (decoding block 5 in this case, since we have 5 decoding blocks), and ${w}_{1}$ would be its respective weight. Note that weights halve with each decoding block, resulting in ${w}_{2}=\displaystyle \frac{1}{2}{w}_{1};{w}_{3}=\displaystyle \frac{1}{4}{w}_{1},$ etc., and are normalized to sum to 1.

Model training used a stochastic gradient descent optimizer with polynomial learning rate decay. Dilated convolution used a dilation factor of 2, and kernel size was 3. Model training was limited to 500000 iterations to prevent model overfitting, with one iteration defined as network training on one training patch. With batch size being one, the number of epochs would therefore equal 500 000 divided by the number of training samples.

### Channel dropout

We employed a novel channel dropout technique inspired by the dropout method widely used in training artificial neural networks. Similar to the dropout method, in which hidden units in certain layers are randomly dropped to reduce overfitting through complex co-adaptations in training, our channel dropout method randomly drops an imaging modality when forming inputs to prevent co-adaptation between different modalities. This technique ensures that the network can learn intrinsic information from each independent modality and any subsequent combination, improving the deployment of a single model trained on multi-modal images on either single-modality or multi-modality images on demand.

**Figure 1.**
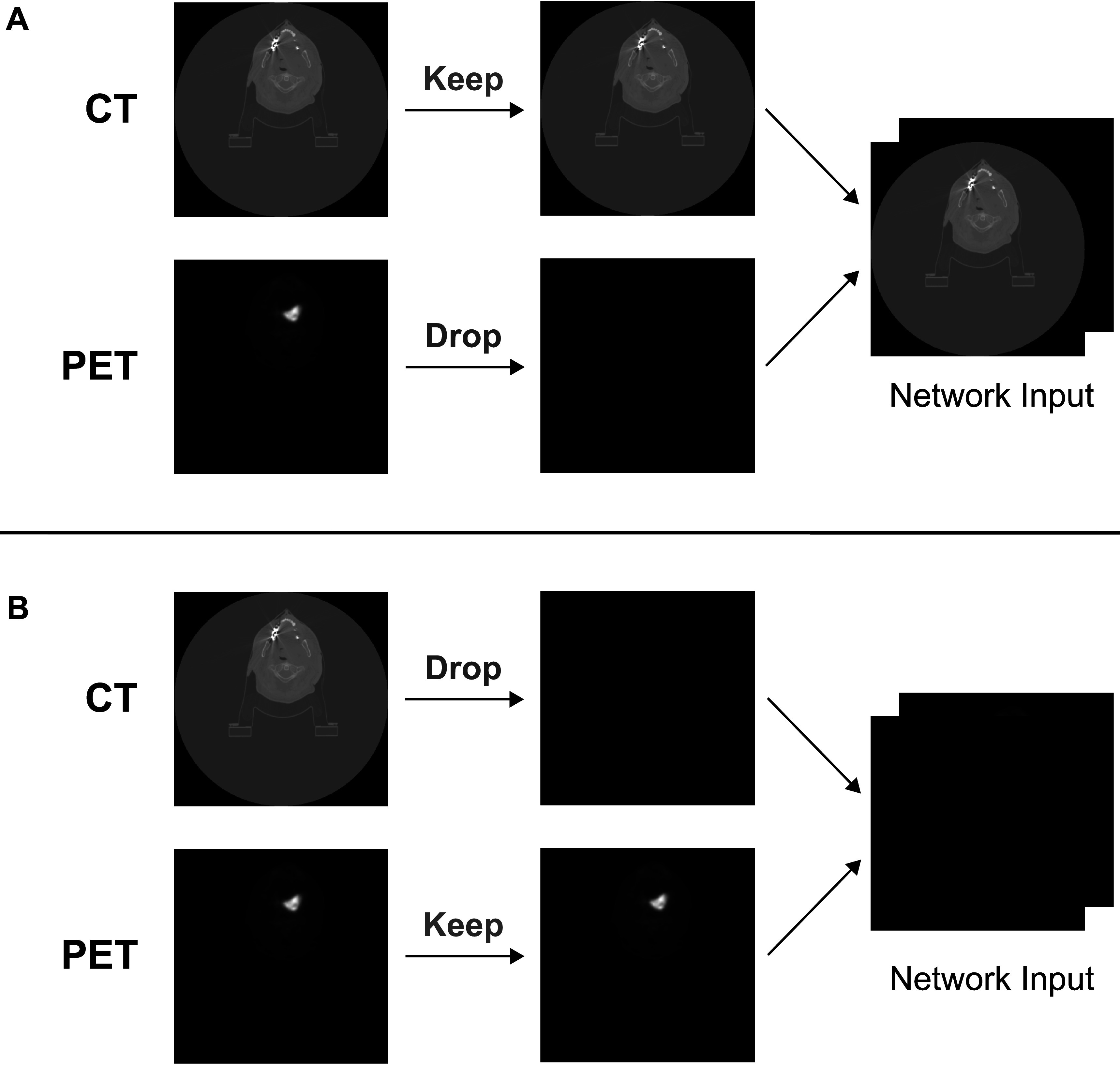
Demonstration of channel dropout preserving the CT image and dropping the PET image (figure 1(A)) or vice versa (figure 1(B)).

Figure 1 illustrates channel dropout implementation. During training, instead of immediately concatenating the CT and PET images into a 4D input image to the network, we randomly decided whether or not to drop one of the modalities by substituting it with an all-zero array. We set the probability of either keeping both modalities versus dropping a modality to 50%. The specific modality (CT or PET) to be dropped was chosen randomly with uniform distribution. We then concatenated the 2 modalities (if one of the modalities was dropped, the all-zero array would be concatenated instead) to form a 4D input image to the network. The rest of the network remained unmodified. By replacing the dropped-out modality with pure background, no additional information was created, preventing any interference with the network. During deployment, the missing modality was similarly simulated by a background image with zero at each voxel.

### Generation of model predictions

We used a sliding window approach, in which individual patches were extracted for prediction before being reassembled to match the original testing images. The sliding window was the same size as the training patch. To optimize merging of predictions in re-assembly, we implemented an overlapping stride of one-fourth of the size of the sliding window, allowing predictions to be averaged.

We took advantage of the symmetric left–right anatomy of the head and neck again to improve test prediction performance. We modeled predictions for both the original patches and left–right flipped versions. The output of the model is a probability array that maps back to the input image, where each value in the array represents the probability of the corresponding voxel in the input image being part of the ROI. The probability maps for the left–right flipped patch were then flipped again (to match the original orientation) and averaged with the probability maps for the original patch to produce an improved output. The averaged probability maps were converted to a binary segmentation mask with a threshold of 0.5. The averaged probability maps produced by models were saved individually to allow for combination in ensemble modeling.

Finally, the binary segmentation masks generated by the model, which are 1 × 1 × 1 mm^3^ isotropic resolution, were resampled back to the original voxel resolution of the input image. For example, if the original image had a voxel resolution of 1 × 1 × 5 mm^3^, the output segmentation masks would be downsampled to 1 × 1 × 5 mm^3^ so that overlay is compatible.

### Ensemble modeling

Ensemble modeling is a technique in which predictions from multiple individual models are synthesized to yield a single higher performing model (Jiang *et al* 2020, Sudharson and Kokil 2020). This allows a single model to leverage distinct model attributes, such as those of dilated (improved capture of global information) and traditional (improved capture of fine details) convolutions. To combine predictions from these models, we averaged the probability maps of the desired models before generating the binary segmentation mask.

## Experiments

### Dataset

The dataset used in this study is from the Medical Image Computing and Computer Assisted Intervention (MICCAI) 2020 ‘HECKTOR’ Challenge for H&N tumor segmentation in PET-CT scans, focusing on oropharyngeal cancers. The dataset includes multi-institutional clinically acquired bimodal H&N scans (FDG-PET and CT) with corresponding annotations of primary gross tumor volume (GTV) for 201 cases. Data originated from HNSCC patients treated at Hôpital Général Juif (HGJ), Centre Hospitalier Universitaire de Sherbrooke (CHUS), Hôpital Maisonneuve-Rosemont (HMR), and Centre Hospitalier de l’Université de Montréal (CHUM). All patients underwent FDG-PET/CT imaging scans within a median of 18 d (range: 6–66) before treatment (Andrearczyk *et al* 2020).

### Model training

80% of the training data (160 cases) was used in the training set, and the remaining 20% (41 cases) was used as the test set. Eight models were trained using the following tunable parameters: (1) different modalities; (2) channel dropout; (3) types of convolutions. Models 1 and 2 were trained with a single input modality of only CT or PET. Models 3–6 were trained with both input modalities but differ in whether they use channel dropout and conventional versus dilated convolution. Finally, Model 7 (referred to as Ensemble 1) is an ensemble between Model 3 and 5, where respective probability maps are averaged before generating a segmentation map, and Model 8 (referred to as Ensemble 2) is an ensemble between Model 4 and 6. Models were trained using the TensorFlow platform with an average training time per model of 72 h (Nvidia Tesla V100 SXM2 GPU, 16 Gb).

### Quantitative evaluation

To evaluate the performance of our segmentation models, we calculated DSC, Hausdorff distance (HD), and volume intraclass correlation coefficient (ICC). DSC was computed using manually-annotated segmentation masks as ground truth and our model’s segmentation output. DSC is calculated as

\begin{eqnarray*}\mathrm{DSC}=\frac{2\left|{\mathrm{X}}{\cap }^{\,}{\mathrm{Y}}\right|}{\left|{\mathrm{X}}\right|+\left|{\mathrm{Y}}\right|},\end{eqnarray*}

in which X represents the ground truth and Y represents the segmented contours from the algorithm. Volume ICC was calculated with the 2-way model from the Interrater Reliability and Agreement (IRR) package in RStudio 2022.07.2 (Gamer *et al* 2010). Metric variability for DSC and HD was calculated by taking the standard deviations of DSC and HD values across the samples in the test set. Metric variability for volume ICC was assessed by calculating the standard deviation of bootstrapped (*n* = 1000) subsets from the test set. Model metrics were compared with a 2-sample z test.

## Results

### Patient characteristics

A total of 201 patients from 4 institutions were enrolled in this study. Patients were predominantly male: 78.2%, 77.8%, 75%, and 69.4% for HGJ, HMR, CHUM, and CHUS, respectively. The average age was (61.6 ± 8.7) years at HGJ, (66.8 ± 9.2) years at HMR, (63.7 ± 9.5) years at CHUM, and (62.4 ± 9.8) years at CHUS. Detailed information can be found in Table 1.

**Table 1.** Description of demographic information and tumor volume.

Institution	HGJ (*n* = 55)	HMR (*n* = 18)	CHUM (*n* = 56)	CHUS (*n* = 72)
Female	12 (21.8)	4 (22.2)	14 (25.0)	22 (30.6)
Age (y)	61.6 ± 8.7	66.8 ± 9.2	63.7 ± 9.5	62.44 ± 9.8
Tumor volume (mm^3^)	14827.7 ± 13063.8	23510.8 ± 27700.3	9847.3 ± 8512.9	14128.3 ± 15920.9

Data is presented as mean ± standard deviation or frequency (percentage). Abbreviations: HGJ = Hôpital général juif, CHUS = Centre hospitalier universitaire de Sherbrooke, HMR = Hôpital Maisonneuve-Rosemont, CHUM = Centre hospitalier de l’Université de Montréal

### Channel dropout creates a model robust to missing modalities

Our results show that when a model expects two modalities (CT and PET), channel dropout drastically improves robustness to missing modalities, especially when PET is missing. Here, we focus on Models 3–6, which were trained on both modalities. Models 3 and 4 both use conventional convolution, but they differ in that Model 4 incorporates channel dropout whereas Model 3 does not. Models 5 and 6 both use dilated convolution, but they differ in that Model 6 incorporates channel dropout whereas Model 5 does not. For conventional convolution, incorporating channel dropout improved DSC to 0.61 from 0.08, HD to 11.67 mm from 38.26 mm, and volume ICC to 0.70 from 0.06 when only CT was available (Model 4 vs Model 3, *p* < 0.001, <0.001, <0.001, respectively). When only PET was available, incorporating channel dropout improved DSC to 0.74 from 0.51, HD to 8.02 mm from 16.36 mm, and volume ICC to 0.95 from 0.45 (Model 4 versus Model 3, *p* < 0.001, <0.001, <0.001, respectively). When both PET and CT were available, most of the metrics showed no significant difference in performance with incorporating channel dropout (Model 4 versus 3; DSC 0.78 versus 0.80 [*p* = 0.522]; HD 7.11 mm versus 6.66 mm [*p* = 0.712]). There was a slight but significant difference in volume ICC (Model 4 versus 3; 0.94 versus 0.97, *p* < 0.001). The robustness that channel dropout adds was also seen with dilated convolution. Detailed model metrics can be found in Table 2 and *p*-value comparisons in Table 3.

**Table 2.** Ablation study comparing model performance among different training parameters. Metrics performed on the test set. Dice similarity coefficient (DSC), Hausdorff distance (HD), and volume intraclass correlation coefficient (ICC) are shown as mean (standard deviation).

	Training parameters			CT only during testing			PET only during testing			CT+PET during testing		
	Training Modality	Channel Dropout	Convolution	DSC	HD (mm)	ICC	DSC	HD (mm)	ICC	DSC	HD (mm)	ICC
Model 1	CT	−	Conventional	0.62 (0.21)	10.94 (7.75)	0.81 (0.08)	−	−	−	−	−	−
Model 2	PET	−	Conventional	−	−	−	0.74 (0.16)	7.77 (5.90)	0.96 (0.03)	−	−	−
Model 3	CT+PET	N	Conventional	0.08 (0.14)	38.26 (17.27)	0.06 (0.07)	0.51 (0.17)	16.36 (11.47)	0.45 (0.07)	0.80 (0.14)	6.66 (5.31)	0.97 (0.02)
Model 4		Y	Conventional	0.61 (0.21)	11.67 (9.04)	0.70 (0.14)	0.74 (0.16)	8.02 (6.80)	0.95 (0.03)	0.78 (0.15)	7.11 (5.67)	0.94 (0.03)
Model 5		N	Dilated	0.07 (0.14)	42.33 (14.54)	0.02 (0.05)	0.55 (0.20)	17.14 (15.46)	0.49 (0.11)	0.79 (0.15)	6.86 (5.94)	0.97 (0.02)
Model 6		Y	Dilated	0.61 (0.22)	14.19 (13.20)	0.84 (0.05)	0.75 (0.14)	7.86 (5.98)	0.93 (0.04)	0.80 (0.13)	5.67 (3.30)	0.96 (0.02)
Ensemble 1	Ensemble between Model 3 and 5			−	−	−	−	−	−	0.80 (0.14)	6.21 (4.76)	0.97 (0.02)
Ensemble 2	Ensemble between Model 4 and 6			−	−	−	−	−	−	0.80 (0.14)	5.68 (3.43)	0.95 (0.03)

**Table 3.** *P*-values of two-sample z tests comparing the performance of models that use channel dropout. *P*-values for the specific metric are shown in their respective column. Significant differences are bolded. Statistical testing for conventional convolution was between Model 3 and 4 whereas dilated convolution was between Model 5 and 6.

	CT only during testing			PET only during testing			CT+PET during testing		
Convolution	DSC	HD	ICC	DSC	HD	ICC	DSC	HD	ICC
Conventional	<0.001	<0.001	<0.001	<0.001	<0.001	<0.001	0.522	0.712	<0.001
Dilated	<0.001	<0.001	<0.001	<0.001	<0.001	<0.001	0.721	0.261	0.119

Abbreviations: DSC = Dice similarity coefficient, HD = Hausdorff distance, ICC = volume intraclass correlation coefficient.

**Figure 2.**
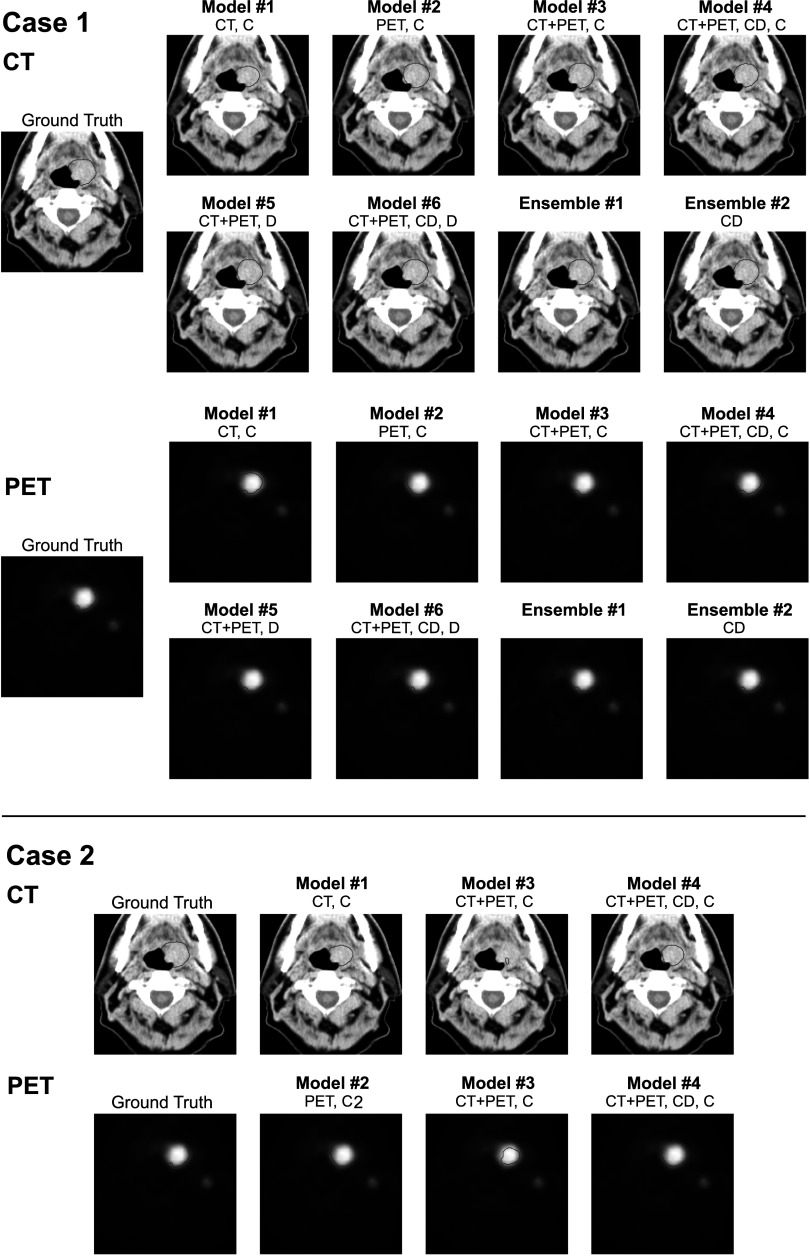
Ground truth and model output when both CT and PET are available (Case 1) and when either CT or PET is missing (Case 2). Model 1 (CT) and Model 2 (PET) were trained and tested on single modalities- model output for Case 1 is overlayed on both modalities for clinical context. CD = Use of Channel Dropout. Convolution: C = Conventional, D = Dilated.

Figure 2 provides a visual representation of these findings, showing ground truth and model output when validated with different modality combinations. In Case 1, models were provided with both CT and PET. All models achieved acceptable segmentation, including those without dropout. In Case 2, models were provided with either CT or PET, modeling a situation with a missing modality. In this case, models trained without channel dropout demonstrated poorer performance.

### Type of convolution does not change performance with channel dropout

There was no significant difference among metrics except for a slight but significant difference in volume ICC when comparing conventional and dilated convolution. Models 4 and 6 both use channel dropout. However, Model 4 uses conventional convolution, while Model 6 uses dilated. When only CT was available, Models 4 and 6 returned DSC of 0.61 versus 0.61 (*p* = 0.947), HD of 11.67 mm versus 14.19 mm (*p* = 0.313), and volume ICC of 0.70 versus 0.84 (*p* < 0.001). When only PET was available, Models 4 and 6 returned DSC of 0.74 versus 0.75 (*p* = 0.840), HD of 8.02 mm versus 7.86 mm (*p* = 0.912), and volume ICC of 0.95 versus 0.93 (*p* = 0.05). When both CT and PET were available, Models 4 and 6 returned DSC of 0.78 versus 0.80 (*p* = 0.437), HD of 7.11 mm versus 5.67 mm (*p* = 0.160), and volume ICC of 0.94 versus 0.96 (*p* = 0.002). With no difference in performance between convolution methods, the rest of the results in text will focus on conventional convolution.

### When only one modality is available, a channel dropout model capable of handling missing sequences performs just as well as a model trained on the single available modality

Comparing Model 2 and 4 demonstrates differences in performance between a single input and channel dropout model when CT is missing and only PET is available. Model 2 was trained only on PET, and Model 4 was trained on both modalities incorporating channel dropout. Metrics between Models 2 and 4 were DSC of 0.74 versus 0.74 (*p* = 0.997), HD of 7.77 mm versus 8.02 mm (*p* = 0.856), and volume ICC of 0.96 versus 0.95 (*p* = 0.054), respectively. When PET was missing and only CT was available, comparisons between Model 1 (single input CT only) and Model 4 were similar. There were no significant differences between DSC (0.62 versus 0.61, *p* = 0.875) and HD (10.94 mm versus 11.67 mm, *p* = 0.692). There was a significant difference in volume ICC, however (0.81 versus 0.70, *p* < 0.001).

### Addition of PET imaging significantly improves segmentation performance

Model 4 was trained on both imaging modalities, incorporating channel dropout, and can therefore demonstrate the individual contribution of each imaging modality to segmentation performance. With only CT imaging, DSC was 0.61, HD 11.67 mm, and volume ICC 0.70. When both PET and CT were used, there was a significant improvement of DSC to 0.78 (*p* < 0.001), HD to 7.11 mm (*p* = 0.006), and volume ICC to 0.94 (*p* < 0.001). Contrastingly, with only PET imaging, the model was able to achieve similar performance to using both inputs with DSC of 0.74, HD 8.02 mm, and volume ICC 0.95 (*p* = 0.314, 0.510, 0.422, respectively).

### There are no differences in performance with an ensemble or single model approach

Ensembles 1 and 2 were specifically created to utilize both convolutions (conventional and dilated), but Ensemble 2 utilizes models that incorporated dropout whereas Ensemble 1 utilizes models that did not. There were no significant differences among DSC, HD, and volume ICC between the ensembles and the individual models comprising them (Ensemble 1 versus Model 3, *p* = 0.945, 0.685, 0.646 [DSC, HD, volume ICC, respectively]; Ensemble 1 versus Model 5, *p* = 0.770, 0.582, 0.626; Ensemble 2 versus Model 4, *p* = 0.557, 0.168, 0.198; Ensemble 2 versus Model 6, *p* = 0.848, 0.987, 0.076). The specific metric values are displayed in Table 2.

## Discussion

In this study, we adopted a novel channel dropout method to develop an automatic H&N tumor segmentation model capable of accurate segmentation despite missing CT and FDG-PET modalities. The main findings from this study were two-fold: (1) segmentation models can handle missing CT and PET imaging modalities with the inclusion of a simple training strategy like channel dropout and (2) a single model using channel dropout can perform just as well as having a separate model for each permutation of available sequences (only CT, only PET, CT and PET). Most deep learning segmentation models are fixed input, meaning that they require all imaging modalities that they were trained on. Having a missing modality (either CT or PET) will inevitably compromise model performance, as demonstrated by the staggering drop in performance of our CT-PET dual input model trained without channel dropout when CT was missing (DSC = 0.51, Model 3) or when PET was missing (DSC = 0.08, Model 3). When the same model was trained with channel dropout, performance was stable when CT was missing (DSC = 0.74, Model 4) and when PET was missing (DSC = 0.61, Model 4). Furthermore, there was no performance compromise with the robustness that channel dropout offers. When both imaging modalities were available, the same model had no statistically significant differences in performance whether it was trained with or without channel dropout (DSC *p* = 0.522, Model 3 versus 4).

The most straightforward alternative to channel dropout in addressing missing modalities is to create a model for every permutation of missing modalities (only CT, only PET, both CT and PET available). When we simplify multiple models into one model capable of missing modalities, there is the question whether performance is being sacrificed for convenience. Models 1 and 2 were single input models, where Model 1 only accepts CT and Model 2 only PET. When a dual input model trained with dropout was only able to use either CT or PET (Model 4), it performed just as well as Model 1 and 2 (DSC *p* = 0.875, 0.997).

One of the concerns with generalizing channel dropout to PET and CT imaging was that a model would depend on synergistic information from both modalities and therefore create complex co-adaptation between modalities that could prevent robustness to missing modalities. Previous studies that successfully use similar methods to channel dropout have largely focused on missing sequences in MRI imaging (van Garderen *et al* 2019, Lau *et al* 2019, La Rosa *et al* 2020). While different sequences may demonstrate different areas of hyper- and hypointensities, the background anatomy present on such imaging is consistent across sequences, allowing for available sequences to compensate for the missing information from unavailable sequences. This is not necessarily true in PET-CT imaging. PET mainly shows areas where radiotracer is concentrated. Therefore, overlay with CT is often done in clinical practice to provide high resolution anatomical context of the ROI. Despite this, our study shows that a model with channel dropout can extract independent information from each modality so that segmentation performance is maintained when a modality is unavailable. When we focus on the CT and PET input model trained with channel dropout (Model 4), the DSC is 0.78 when both modalities are available. When only CT is available, the DSC only falls to 0.61. When only PET is available, channel dropout accounts for missing information well enough to the point there is no significant difference in performance (*p* = 0.314).

This raises a separate question of whether having solely PET imaging is sufficient for accurate segmentation of H&N tumors by deep learning models. Our studies suggest that this may be true. However, this is task specific. Segmentation of different tumors that uptake radiotracer differently or have more prominent characteristics on CT may demonstrate statistically significant performance enhancement by having both imaging modalities. Furthermore, PET is ultimately a harder imaging study to obtain than CT, requiring a specific diet before image acquisition and use of a specific radiotracer. Therefore, a model reliant on only PET input may encounter more frequent issues with missing imaging modalities.

We also experimented with different convolution methods and ensemble strategies to augment segmentation performance. Dilated convolution has been proposed to increase the receptive field of a network, helping to extract more global and higher-level semantic features (You *et al* 2022). We did not observe a significant improvement in performance between dilated and conventional convolution methods (DSC *p* = 0.947). Furthermore, our ensemble strategy that combined one model using conventional convolution with another using dilated convolution also did not demonstrate significant differences in performance from a single model strategy (average *p*-value between DSC of Ensemble 1 and its individual models is 0.858; average *p*-value between DSC of Ensemble 2 and its individual models is 0.703).

To evaluate model performance, we used three metrics: DSC, HD, and volume ICC. This allowed us to see how model segmentation output compares to ground truth with respect to segmentation overlap, maximum distance between segmentations, and segmentation size. In general, comparisons among DSCs, HDs, or volume ICCs reached the same conclusions in determining whether a model was superior to another. However, there were certain instances where comparisons in DSC or HD demonstrated no statistical significance whereas volume ICC showed a slight but significant difference (e.g. Model 4 versus 3 with CT and PET, absolute difference DSC = 0.02, *p* = 0.522; absolute difference HD = 0.45 mm, *p* = 0.712; absolute difference volume ICC = 0.03, *p* < 0.001). The higher sensitivity to statistical difference with volume ICC is to be expected, as it compares differences between two sets of samples when evaluating volume correlation whereas DSC and HD compares differences between two individual samples. Thus, the standard deviation of volume ICC would inherently be much smaller than those of DSC and HD, leading to instances of statistically significant but clinically non-significant differences in volume ICC.

Multiple approaches to tumor segmentation using PET and CT imaging have been proposed that either support our findings or suggest an alternative network architecture that we may employ in the future. Zhao *et al* also demonstrated that using both PET and CT for lung cancer segmentation (DSC = 0.85) outperforms CT alone (DSC = 0.76) but performs similarly to PET alone (DSC = 0.83) (Zhao *et al* 2018). Song *et al* proposed another method for lung and H&N tumor co-segmentation in both PET and CT images, using a Markov random field (MRF) model approach. Their results corroborate the importance of PET as their proposed co-segmentation method had a DSC of 0.81 compared to a DSC of 0.66 with PET only and DSC of 0.48 with CT only (Song *et al* 2013). Andrearczyk *et al* had the most success in H&N segmentation with late fusion of PET and CT imaging by inputting axial slices in two separate 2D V-Nets, one for CT and another for PET, and averaging the probability maps together. (Andrearczyk *et al* 2020) They built a 3D segmentation slice by slice to achieve a DSC of 0.606. Guo *et al* employed a 3D Dense-Net to achieve a DSC of 0.71 with PET/CT, compared to 0.64 with PET and 0.31 with CT alone (Guo *et al* 2019). They also employed a 3D U-Net on PET/CT, which achieved a comparable DSC of 0.69. Groendahl *et al* compared 2D U-Net CNNs to PET thresholding methods and classical machine learning classifiers in a large HNSCC cohort (Groendahl *et al* 2021). Their results support using deep learning models, as PET/CT input CNNs achieved the greatest performance (DSC = 0.74) compared to PET thresholding (DSC = 0.62) and classical machine learning (DSC = 0.66). Similar to our results, several of these studies observed higher performance on PET than CT when exploring single modalities.

The dataset used in this study is from the MICCAI 2020 ‘HECKTOR’ Challenge, which focused on automatic segmentation of H&N oropharyngeal primary tumors in combined FDG-PET and CT imaging (Oreiller *et al* 2022). The training set consisted of 201 cases from four different centers and methods were tested on 53 cases from a fifth center. All 64 teams registered to participate in the challenge used U-Net based architectures, with preprocessing, normalization, data augmentation, and ensembling playing important roles in performance. The testing set from the original dataset was not used in this study since ground-truth values were not readily accessible, providing no means of evaluating model performance. As a solution, we divided the 201 provided cases into 160 training cases and 41 testing cases for our models. Thus, our results may not be directly comparable to those from the challenge due to our smaller training dataset and different testing set. The highest performing participant employed a U-Net architecture with Squeeze-and-Excitation (SE) Normalization layers and constructed an ensemble of eight models trained and tested on different splits of the training set (Iantsen et al 2021). Chen *et al* iteratively refined segmentation with three U-Nets, using features and results of upstream models to inform subsequent models. They achieved a DSC of 0.72 (Chen *et al* 2021).

There are several limitations in this study. We evaluated our channel dropout model’s robustness to missing modalities with edge cases (either all CT or all PET images were dropped from test cases). However, in clinical practice, useable or available sequences will certainly make up the majority of cases. As mentioned in the introduction, artifact can be present in many cases, but whether or not this artifact is significant enough to be dropped is unclear. Further investigation of the robustness of our channel dropout model in clinical practice should be conducted. Furthermore, our experiments assign a certain imaging modality with a binary state: available or missing. In clinical practice, this dichotomy is not so clear. For example, motion artifact outside of the ROI may not render an image unusable. Future studies incorporating a pipeline that can automatically evaluate the utility of an imaging modality can further the applicability of our channel dropout models.

## Conclusion

We demonstrate that a channel dropout technique can create a H&N tumor segmentation model robust to missing PET or CT imaging modalities. This allows us to combine the advantages of multiple modalities without deploying multiple models for each permutation of missing modalities.

## Data Availability

The data cannot be made publicly available upon publication because they are owned by a third party and the terms of use prevent public distribution. The data that support the findings of this study are available upon reasonable request from the authors.

## Funding information

This project has been funded in whole or in part with federal funds from the National Cancer Institute, National Institutes of Health, and Department of Health and Human Services under Contract No. 75N91020C00048. Funding was also received from the Huxiang High-level Talent Gathering Project (2021RC5003) and Ministry of Science and Technology Foreign High-End Experts Introduction Project (G2022161002L).

## Ethical statement

The authors declare no conflicts of interest. This study used a public dataset, and informed consent was not applicable.

## References

[pmbaccac9bib1] Andrearczyk V, Oreiller V, Vallières M, Castelli J, Elhalawani H, Jreige M, Boughdad S, Prior J O, Depeursinge A (2020). Automatic segmentation of head and neck tumors and nodal metastases in PET-CT scans. Medical imaging with deep learning. PMLR.

[pmbaccac9bib2] Bellver M, Maninis K-K, Pont-Tuset J, Giró-I-Nieto X, Torres J, Van Gool L (2017). arXiv.

[pmbaccac9bib3] Bibb R, Eggbeer D, Paterson A (2015). Medical Modelling : the Application of Advanced Design and Rapid Prototyping Techniques in Medicine.

[pmbaccac9bib4] Blanc-Durand P, Van Der Gucht A, Schaefer N, Itti E, Prior J O (2018). Automatic lesion detection and segmentation of 18F-FET PET in gliomas: a full 3D U-Net convolutional neural network study. PLoS One.

[pmbaccac9bib5] Blodgett T M, Mehta A S, Mehta A S, Laymon C M, Carney J, Townsend D W (2011). PET/CT artifacts. Clin Imaging.

[pmbaccac9bib6] Brosch T, Tang L Y W, Yoo Y, Li D K B, Traboulsee A, Tam R (2016). Deep 3D convolutional encoder networks with shortcuts for multiscale feature integration applied to multiple sclerosis lesion segmentation. IEEE Trans. Med. Imaging.

[pmbaccac9bib7] Chen H, Chen H, Wang L (2021). Iteratively refine the segmentation of head and neck tumor in FDG-PET and CT images. Cham. Springer Int. Publ..

[pmbaccac9bib8] Dou Q, Yu L, Chen H, Jin Y, Yang X, Qin J, Heng P-A (2017). 3D deeply supervised network for automated segmentation of volumetric medical images. Med. Image Anal..

[pmbaccac9bib9] Feng Y, Pan H, Meyer C, Feng X (2018). arXiv.

[pmbaccac9bib10] Gamer M, Lemon J, Singh I (2010). irr: Various Coefficients of Interrater Reliability and Agreement.

[pmbaccac9bib11] Van Garderen K, Smits M, Klein S (2019). Multi-modal segmentation with missing MR sequences using pre-trained fusion networks. DART MIL3ID 2019 2019. Lecture Notes in Computer Science.

[pmbaccac9bib12] Girshick R (2015). Fast r-cnn.

[pmbaccac9bib13] Goel R, Moore W, Sumer B, Khan S, Sher D, Subramaniam R M (2017). Clinical practice in PET/CT for the management of head and neck squamous cell cancer. AJR Am. J. Roentgenol..

[pmbaccac9bib14] Gould K L, Pan T, Loghin C, Johnson N P, Guha A, Sdringola S (2007). Frequent diagnostic errors in cardiac PET/CT due to misregistration of CT attenuation and emission PET images: a definitive analysis of causes, consequences, and corrections. J. Nucl. Med..

[pmbaccac9bib15] Groendahl A R (2021). A comparison of methods for fully automatic segmentation of tumors and involved nodes in PET/CT of head and neck cancers. Phys. Med. Biol..

[pmbaccac9bib16] Gudi S (2017). Interobserver variability in the delineation of gross tumour volume and specified organs-at-risk during IMRT for head and neck cancers and the impact of FDG-PET/CT on such variability at the primary site. J. Med. Imaging Radiat. Sci..

[pmbaccac9bib17] Guo Z, Guo N, Gong K, Zhong S, Li Q (2019). Gross tumor volume segmentation for head and neck cancer radiotherapy using deep dense multi-modality network. Phys. Med. Biol..

[pmbaccac9bib18] Hinton G E, Srivastava N, Krizhevsky A, Sutskever I, Salakhutdinov R R (2012). Improving neural networks by preventing co-adaptation of feature detectors. http://arxiv.org/abs/1207.0580.

[pmbaccac9bib19] Hussain S, Anwar S M, Majid M (2018). Segmentation of glioma tumors in brain using deep convolutional neural network. Neurocomputing.

[pmbaccac9bib20] Hussain Z, Gimenez F, Yi D, Rubin D (2017). Differential data augmentation techniques for medical imaging classification tasks. AMIA Annu. Symp. Proc..

[pmbaccac9bib21] Iantsen A, Visvikis D, Hatt M (2021). Squeeze-and-excitation normalization for automated delineation of head and neck primary tumors in combined PET and CT images.. Head and Neck Tumor Segmentation: First Challenge, HECKTOR 2020, Held in Conjunction with MICCAI 2020, Proc. 1, 2021.

[pmbaccac9bib22] Isensee F, Jaeger P F, Kohl S A A, Petersen J, Maier-Hein K H (2021). nnU-Net: a self-configuring method for deep learning-based biomedical image segmentation. Nat. Methods.

[pmbaccac9bib23] Jafari M H, Karimi N, Nasr-Esfahani E, Samavi S, Soroushmehr S M R, Ward K, Najarian K (2016). Skin lesion segmentation in clinical images using deep learning.

[pmbaccac9bib24] Jiang J, Yu Y, Wang Z, Tang S, Hu R, Ma J (2020). Ensemble super-resolution with a reference dataset. IEEE Trans. Cybern..

[pmbaccac9bib25] Kawauchi K, Furuya S, Hirata K, Katoh C, Manabe O, Kobayashi K, Watanabe S, Shiga T (2020). A convolutional neural network-based system to classify patients using FDG PET/CT examinations. BMC Cancer.

[pmbaccac9bib26] Kumar A, Fulham M, Feng D, Kim J (2020). Co-learning feature fusion maps from PET-CT images of lung cancer. IEEE Trans. Med. Imaging.

[pmbaccac9bib27] Langer A (2010). A systematic review of PET and PET/CT in oncology: a way to personalize cancer treatment in a cost-effective manner?. BMC Health Serv. Res..

[pmbaccac9bib28] Lau K, Adler J, Sjölund J (2019). A unified representation network for segmentation with missing modalities. http://arxiv.org/abs/1908.06683.

[pmbaccac9bib29] Lautamäki R, Brown T L, Merrill J, Bengel F M (2008). CT-based attenuation correction in (82)Rb-myocardial perfusion PET-CT: incidence of misalignment and effect on regional tracer distribution. Eur. J. Nucl. Med. Mol. Imaging.

[pmbaccac9bib30] Lecun Y, Bengio Y, Hinton G (2015). Deep learning. Nature.

[pmbaccac9bib31] Lewis-Jones H, Colley S, Gibson D (2016). Imaging in head and neck cancer: United Kingdom National Multidisciplinary Guidelines. J. Laryngol Otol.

[pmbaccac9bib32] Li L, Zhao X, Lu W, Tan S (2020). Deep learning for variational multimodality tumor segmentation in PET/CT. Neurocomputing.

[pmbaccac9bib33] Li X, Dou Q, Chen H, Fu C-W, Qi X, Belavý D L, Armbrecht G, Felsenberg D, Zheng G, Heng P-A (2018). 3D multi-scale FCN with random modality voxel dropout learning for intervertebral disc localization and segmentation from multi-modality MR Images. Med. Image Anal..

[pmbaccac9bib34] Long J, Shelhamer E, Darrell T (2015). Fully convolutional networks for semantic segmentation.

[pmbaccac9bib35] Moe Y M, Groendahl A R, Mulstad M, Tomic O, Indahl U, Dale E, Malinen E, Futsaether C M (2019). Deep learning for automatic tumour segmentation in PET/CT images of patients with head and neck cancers. http://arxiv.org/abs/1908.00841.

[pmbaccac9bib36] Oreiller V (2022). Head and neck tumor segmentation in PET/CT: the hecktor challenge. Med. Image Anal..

[pmbaccac9bib37] Parker J A, Kenyon R V, Troxel D E (1983). Comparison of interpolating methods for image resampling. IEEE Trans. Med. Imaging.

[pmbaccac9bib38] Protonotarios N E, Katsamenis I, Sykiotis S, Dikaios N, Kastis G A, Chatziioannou S N, Metaxas M, Doulamis N, Doulamis A (2022). A few-shot U-Net deep learning model for lung cancer lesion segmentation via PET/CT imaging. Biomed. Phys. Eng. Express.

[pmbaccac9bib39] Ronneberger O, Fischer P, Brox T (2015). U-net: convolutional networks for biomedical image segmentation. medical image computing and computer-assisted intervention–MICCAI 2015.

[pmbaccac9bib40] La Rosa F, Beck E S, Abdulkadir A, Thiran J, Reich D S, Sati P, Cuadra M B (2020). Automated detection of cortical lesions in multiple sclerosis patients with 7T MRI. MICCAI 2020. MICCAI 2020. Lecture Notes in Computer Science.

[pmbaccac9bib41] Send T, Kreppel B, Gaertner F C, Bundschuh R A, Strunk H, Bootz F, Essler M (2017). PET-CT bei Karzinomen im Kopf-Hals-Bereich. HNO.

[pmbaccac9bib42] Sharma N, Aggarwal L M (2010). Automated medical image segmentation techniques. J. Med. Phys..

[pmbaccac9bib43] Song Q, Bai J, Han D, Bhatia S, Sun W, Rockey W, Bayouth J E, Buatti J M, Wu X (2013). Optimal co-segmentation of tumor in PET-CT images with context information. IEEE Trans. Med. Imaging.

[pmbaccac9bib44] Srivastava N, Hinton G, Krizhevsky A, Sutskever I, Salakhutdinov R (2014). Dropout: a simple way to prevent neural networks from overfitting. J. Mach. learn. res..

[pmbaccac9bib45] Sudharson S, Kokil P (2020). An ensemble of deep neural networks for kidney ultrasound image classification. Comput. Methods Programs Biomed..

[pmbaccac9bib46] Sureshbabu W, Mawlawi O (2005). PET/CT imaging artifacts. J. Nucl. Med. Technol.,.

[pmbaccac9bib47] Van Den Wyngaert T, De Schepper S, Carp L (2020). Quality assessment in FDG-PET/CT imaging of head-and-neck cancer: one home run is better than two doubles. Frontiers Oncol..

[pmbaccac9bib48] You G-R, Shiue Y-R, Su C-T, Huang Q-L (2022). Enhancing ensemble diversity based on multiscale dilated convolution in image classification. Inf. Sci..

[pmbaccac9bib49] Zhao X, Li L, Lu W, Tan S (2018). Tumor co-segmentation in PET/CT using multi-modality fully convolutional neural network. Phys. Med. Biol..

[pmbaccac9bib50] Zhong Y, Yang Y, Fang Y, Wang J, Hu W (2021). A preliminary experience of implementing deep-learning based auto-segmentation in head and neck cancer: a study on real-world clinical cases. Frontiers Oncol..

